# Analysis of the Attitude of Spanish Physical Education Teachers towards Students with Disabilities in Extremadura

**DOI:** 10.3390/ijerph19095043

**Published:** 2022-04-21

**Authors:** Jorge Rojo-Ramos, María José González-Becerra, Eugenio Merellano-Navarro, Santiago Gomez-Paniagua, José Carmelo Adsuar

**Affiliations:** 1 Motricity and Education (HEME) Research Group, Department of Health, Economy, University of Extremadura, Avda. de la Universidad s/n, 10003 Cáceres, Spain; jorgerr@unex.es (J.R.-R.); jadssal@unex.es (J.C.A.); 2 BioẼrgon Research Group, University of Extremadura, 10003 Cáceres, Spain; mgonzalexd@alumnos.unex.es; 3 Grupo de Investigación EFISAL, Universidad Autónoma de Chile, Talca 3460000, Chile

**Keywords:** physical education, disability, teacher attitude

## Abstract

Inclusive education aims to eliminate barriers in the participation and performance of students, considering their diversity. In this sense, there is a regulation that governs the educational response, being different for each region. Therefore, this study aims to know the attitude of Physical Education teachers of different educational stages in Spain toward students with disabilities. A questionnaire was administered to 272 Physical Education teachers from public schools in a region of Spain. The Mann–Whitney U test was used to determine the relationships between items and dimensions according to sex or center location, and Spearman’s Rho was used to analyze the relationship between dimensions and years of experience. The main results showed that teachers do not feel prepared in terms of training, resources, and infrastructure, although they consider that the integration of students with disabilities in regular classes is beneficial for them.

## 1. Introduction

Today it is increasingly common to see a person with a disability as a person with the same rights and opportunities as any other; however, some social stigmas still prevail about this population due to old beliefs, in spite of the visualization efforts of institutions [1].

The World Health Organization (WHO) understands disability as the interaction between people who have a health problem and personal and environmental factors and estimates that 15% of the world’s population lives with some type of disability, a figure that is increasing dramatically [2]. In Spain, about 1,840,700 people, 6.12% of the population, suffer from some type of disability and must deal with the barriers that society places on them, which are not as easily overcome as those imposed by their bodies. The Olivenza report, published in 2018, also reveals that only one-third of this group is integrated into the labor market and that the school dropout rate is 43.2% [3].

Inclusive education promotes the removal of barriers to participation and achievement for all learners, considering the diversity of their needs, abilities, and particularities, and eliminating all forms of discrimination in learning [4]. Implementing this educational model can be quite challenging, as a diversity of learners is the norm today. Organizations such as the United Nations Educational, Scientific and Cultural Organization (UNESCO) or the United Nations (UN) emphasize the urgent need to ensure equal, inclusive, quality education and the promotion of opportunities for all human beings [5,6]. In the same vein, others join the proposal, calling for the promotion of attention to diversity of any kind (ethnicity, culture, gender) to ensure that all students have access to inclusive and quality education [5,7]. Data from the Survey on Disabilities, Impairments and Health Status [8] show that the percentage of the non-disabled population aged 10 years or more who are illiterate is 2%, which is 14% in the case of people with disabilities. In addition, 32% of the non-disabled population has higher education, while only 9% of the disabled population has it [8].

The Law on Attention to Diversity has been evolving in Spain over the years, and measures are increasingly being adopted to ensure the full inclusion of students with special educational needs. The natural variability that exists in educational groups makes it necessary to adopt measures to address this diversity, understanding it as an enriching element and not as a factor of inequality [9]. The law in charge of regulating the educational system and, which at the same time, emphasizes the value of inclusion following the principles of the Convention on the Rights of Persons with Disabilities [10], is the Organic Law for the Improvement of Educational Quality (LOMLOE) 8/2013 [11]. In addition, its own educational regulations must be developed in each region, being in the case of Extremadura, Decree 228/2014, the one that regulates the educational response to diversity [12].

However, truly achieving inclusive education involves overcoming different obstacles. Negative attitudes are one of the main barriers that limit the participation and full inclusion of people with disabilities in society, experiencing stereotypes based on prejudices and false beliefs that label this group as dependent, inferior, antisocial, or incapable [13,14]. These labels constitute barriers to education, employment, health care, and social participation [15]. Such attitudes move people with disabilities closer to a restriction in behaviors and opportunities and consolidate as a real obstacle to social inclusion [16,17]. Fortunately, there are studies that show that attitudes towards people with disabilities can be modified, but they require the continued implementation of awareness programs based on attitude change techniques [15,16,18,19,20]. Likewise, physical activity-sport is a promising tool to improve the inclusion and participation of students with different abilities among schoolchildren.

Therefore, if we talk about disability in the school context, we must consider not only physical, cognitive, or sensory impairments but also the social context in which they must interact [21], since being able to include students with disabilities in Physical Education (PE) classes while attending to the rest of the student body is a challenge for teachers [22]. Furthermore, what is the role of Physical Education for students with disabilities? Participating in PE sessions is a right that protects every student, and non-participation cannot be excused by any type of special educational support need, implying inclusion in the learning process [23,24]. The fundamental task of the PE teacher should be to contribute to the integral education and emancipation of their students, favoring a change in behavior so that they socialize with each other. In addition, it should favor the process of school socialization, increase their self-esteem, and be a source of positive values such as self-control, self-improvement, cooperation, discipline, companionship, etc. [25]. The attitudes and self-efficacy of teachers in this area are considered essential for the inclusion process, favoring or limiting learning and participation in classes [26,27,28].

On the other hand, the concept of “learning barriers” is applied to the analysis of the inadequate educational response that can be offered by the school, the organization, or the teachers. If we analyze what these barriers may be, according to Ríos Hernández [29]:

▪ Infrastructural conditioning factors: the scarcity of economic resources or difficulty of accessibility.
▪ Social conditioning factors: lack of knowledge of the population.
▪ Conditions of students with disabilities: self-marginalization, need for attention, difficulty in social relations, low level of self-acceptance.
▪ Teaching practice conditioning factors: undervaluation of PE, shortage of teacher training, ideology, negative experiences, family factor, class group, or diagnosis of the person.

Therefore, this study aims to analyze the attitude toward students with disabilities of Physical Education teachers in the region of Extremadura at the Primary, Secondary, and Baccalaureate stages to reinforce knowledge in this field, pointing out those differences between sex and center location in addition to assessing the reliability of the dimensions included in the questionnaire. Thus, this research characterizes the current attitudes of teachers towards students with disabilities, which allows the development of various lines of action for these students by public institutions and the detection of the training needs of teachers in this context.

## 2. Materials and Methods

### 2.1. Participants

The study sample consisted of 272 physical education teachers (Table 1) from both rural and urban public schools in Extremadura (Spain). In the sample, 56.5% (*n* = 154) were men and 43.4% (*n* = 118) were women, the median age was 44 years. All participants were selected following a non-probability sampling method based on convenience sampling [30].

### 2.2. Instruments and Measures

To obtain the sociodemographic data of the sample, a questionnaire was prepared with 6 sociodemographic questions (sex, studies, school environment, teaching, age, and years of teaching experience).

The questionnaire for teachers’ attitudes towards students with special educational needs derived from disability was used. It was designed and validated by García and his colleagues [31], developing both exploratory and confirmatory factor analysis in a population of teachers. This questionnaire was initially developed in Spanish by Franco et al. [32], considering the attitudes of the teachers and students towards disability. Subsequently, it was adapted by Doménech and his colleagues [33] to take into account only those attitudes that involve the teachers.

The instrument was composed of 22 items grouped into five dimensions: social development (items 21, 18, 17.5, and 3), classroom climate (items 22, 20, 19, and 2), training and resources (items 15, 14, 13, 12, 11, and 10), performance (items 16, 4 and 1), and responsibility (items 9, 8, 7, and 6). Each of the items has a Likert-type response format of 1 to 5, with 1 being “strongly disagree” and 5 being “strongly agree”. The authors reported moderate reliability coefficients for the training and resources (α = 0.78), classroom climate (α = 0.78), and social development (α = 0.83) dimensions and particularly low for the responsibility (α = 0.58) and performance (α = 0.42) dimensions [31].

### 2.3. Procedures

It was decided to use the Google Forms application to develop an e-questionnaire that included the sociodemographic questions and the questionnaire for the evaluation and interpretation of teachers’ attitudes towards students with disabilities, as a way to save costs and facilitate the delivery of the questionnaires to the participants [34]. Data collection was carried out between April and December 2021.

To access the sample, the database of public educational centers of the Autonomous Community of Extremadura (Spain) available at (https://ciudadano.gobex.es/ciudadano-portlet/printpdf/pdf?typepdf=3443&idDirectorio=775 (accessed on 1 February 2020)) was accessed, and the contact details of the schools and institutes in which primary or secondary education was taught were selected.

To administer the instrument, an e-mail was sent to the physical education teachers and professors of all the selected centers informing them of the objective of the study, informed consent, and providing a URL link to access the form. The response rate was 54.4% percent, considering that there are 500 PE teachers in the region.

### 2.4. Statistical Analysis

The analysis of the data collected was carried out with the Statistical Package for Social Sciences (SPSS) version 24.0 for MAC. First, Cronbach’s Alpha was used to calculate the reliability for each of the dimensions of the instrument.

To analyze whether the variables complied with the assumption of normality, the Kolmogorov Smirnov test was used, which indicated that this assumption was not met, so it was decided to use nonparametric tests. The Mann–Whitney U test was used to analyze the differences between the different items and dimensions of the questionnaire according to sex and center location (Table 2 and Table 3) and Spearman’s Rho test was used to analyze the relationship between each of the dimensions and the variable age (Table 4).

## 3. Results

Table 1 shows the characterization of the sample, presenting the frequency distribution according to sex, studies, school environment, teaching, age, and years of teaching experience.

Table 2 shows the descriptive data for each of the questionnaire items and the differences between each item according to sex and center location. The Mann–Whitney U test was used to find the differences. The scores for each item are presented as the median and interquartile range (IQR).

Table 3 presents the scores obtained in each of the dimensions of the questionnaire according to sex and center location.

As a function of gender, statistically significant differences were found in the dimensions “social development” and “performance”, with higher scores for males than for females. As a function of school location, statistically significant differences were found in the dimensions “social development”, “classroom climate”, and “achievement”. Urban schools scored higher in the “social development” and “achievement” dimensions and rural schools in the “classroom climate” dimension.

Table 4 shows the relationship between each of the dimensions and age using Spearman’s Rho test.

Finally, the reliability results for each of the dimensions of the questionnaire were a1 = 0.80; a2 = 0.75; a3= 0.74; a4 = 0.71; a5 = 0.73; all values being satisfactory above 0.70 according to [35].

## 4. Discussion

The present study was carried out to learn about the attitude of PE teachers towards disability in the classroom throughout different stages, explaining the differences according to sex and the location of the educational center. The attitudes of PE teachers in their classes are going to be fundamental for the concept of inclusive education to be possible. To this end, a questionnaire was passed around that collects questions on whether there is social development, what the classroom climate experience is, whether the teachers have adequate training and the necessary resources, good performance, and the responsibility required.

The items in Table 2 (3, 5, 17, 18, 21) that refer to the dimension of social development as a process that leads to the improvement of the living conditions of the population receive the highest scores both if we analyze by sex and by location of the center, which means that men and women from rural and urban environments strongly agree with the benefits of integration in education. Other research coincides with these results in that the organization of PE classes offers an opportunity for participation and promotion of coexistence for all students [36] and that smaller classes would positively affect teachers’ attitudes and self-efficacy [37]. In all the items, there are significant differences by sex (*p*-value < 0.01), which means that men and women do not think equally about the role of integration in the classroom [38], except for when it is said that the integration of students with disabilities will promote their social independence. In this case, both sexes agree. If we look for significant differences in the responses according to the location of the center, there are only differences between teachers in urban and rural centers when talking about the positive effect of integration on emotional development and that it favors the preparation of students to live in society, two statements in which the responses are conditioned by the location of the educational center where classes are taught [39].

When it comes to the climate in classrooms with students with disabilities (items 2, 19, 20, 22 of Table 2), the teachers’ responses are more disparate. All teachers point out that maintaining order and discipline is not at all easy, as also pointed out by previous studies [40,41], something on which teachers from both urban and rural centers agree. They agree that students with disabilities behave well in class, but men and women do not feel the same way (items 19 and 20) [42].

If we now examine items 10 to 15 of Table 2 that deal with whether the centers have adequate professionals and resources, we observe average scores in those that correspond to adequate training, specialized personnel, and the incorporation of support, which means that the teachers think that it is not enough. In this line, numerous publications [43,44,45] show that the initial preparation is insufficient to develop competencies related to inclusive education, so they should attend preparation courses to stop feeling that they are not fully prepared to face this task. In addition, there are significant differences (*p*-value < 0.01) in the responses according to sex when referring to the training offered by the center (item 10) or if it contemplates the incorporation of support (item 12), with women being the ones who value it more positively [46]. The location of the educational center only influences the differences in the response to the training offered by the center, being higher in urban than in rural centers [47]. Teachers in urban and rural centers do not agree that the use of specialized materials benefits students with disabilities [48].

In the questions of the questionnaire related to student performance (items 1, 4, and 16 of Table 2), teachers gave, in general, average scores, but let us analyze the answers one by one to understand them. Most teachers agree that for a student with a disability being in a regular classroom presents a challenge that helps to stimulate academic development. This result coincides with those of An et al. [49], who state that students with disabilities are engaged in PE because they find an opportunity to receive a greater number and diversity of activities. The answers given in this case have depended on gender and center location since significant differences are found between the two (*p*-value < 0.01). In the question of whether the student with disabilities can face challenges with equality to the rest of the students, they neither agree nor disagree, and furthermore, all teachers, regardless of sex or location of the center, think the same since no significant differences are found. Finally, teachers think that the extra attention required by students with disabilities is, in many cases, detrimental to the rest. The responses are different depending on gender (item 1 and 16), with women thinking that this is not the case and men the opposite [50]. With respect to the location of the center, in urban environments, they think that it will not be to the detriment of the rest of the students, while in rural environments, they think it will [51].

Finally, and to conclude Table 2, we will analyze the response to items 6 to 9, which refer to the responsibility of teachers in the education of students with disabilities. Teachers agree that the training of students with disabilities is not the responsibility of specialists but of classroom tutors, who should oversee making curricular adaptations with the help of specialists and area teachers. Other authors such as also highlighted the benefits of including a support teacher [45,52,53], such as special education teachers. The responses according to sex do not show significant differences; therefore, men and women agree on the answers. On the other hand, the location of the center is a reason for differences in the responses (*p*-value < 0.01) since teachers in urban centers do not agree as much with the responsibility of the tutors as teachers in rural centers [54].

Table 3 shows each of the dimensions that have been analyzed in the questionnaire and that group the different items. The results are very similar to what we have analyzed previously, observing here more clearly that there are significant differences in the social development and performance dimensions both by sex and by location of the center (*p*-value < 0.01), with higher scores being obtained by men, when we talk about sex, and by teachers from urban centers if we refer to the location of the center. In addition, the responses for the classroom climate dimension were different according to the location of the center, being higher in rural centers compared to those located in urban environments.

Finally, Table 4 shows the correlation between the different dimensions according to years of teaching experience. It is shown that the correlation and magnitude are significant only in the responsibility dimension (*p*-value < 0.01 **, *p* = −0.50), so it can be said that the score received by this dimension will vary negatively as years of teacher’s experience increase. In the rest of the dimensions, there are no significant differences, so they will not vary according to the years of experience. This research presents various limitations, as we did not ask whether teachers attended specific courses on inclusive education, and we studied the opinion of teachers in only one region, affecting different sociodemographic and cultural issues. Additionally, the selection bias could further narrow the sample according to more specific characteristics of the teaching staff, as priority was given to obtaining a larger sample.

## 5. Conclusions

The PE teachers of the Primary, Secondary, and Baccalaureate stages agree with the positive effects of integration on the emotional development that favors the preparation of students with disabilities to live in society. In addition, they believe that their social image improves through interaction with the group and that their behavior in class is good, but men and women think differently. Regarding the training of professionals, resources, and infrastructures, teachers think that they are not sufficient since they do not have adequate training, specialized personnel, or the resources they consider necessary.

Teachers agree with the benefits for a student with a disability to be in an ordinary classroom as a stimulus for his or her academic development, but also that it can be a disadvantage to pay sufficient attention to the rest of the students. In addition, they believe that the training of students is the responsibility of the tutors, who should oversee curricular adaptations with the help of specialists and other teachers in the area. It is important to highlight the relationship found between the years of teaching experience and the change in thinking about who is responsible for curricular adaptations.

Consequently, both teaching professionals at any level as well as teachers still in training, the members of public and private institutions involved in training and education, as well as the parents of the students could beneficiate from the study’s findings to better understand the consequences, implications, and importance of these attitudes.

Finally, it can be proposed for future studies to extend it to other territories to be able to compare what happens throughout the national territory. Furthermore, it would be interesting to consider specific training in educational inclusion as a conditioning variable, as well as the age and the years of experience. Moreover, regression analyses should be implemented between variables and the questionnaire’s dimensions to understand the cause-effect relationships between them. Additionally, evaluating future teachers’ perspectives and including private schools could be considered future lines of research.

## Figures and Tables

**Table 1:** Frequency distribution of the sample (N = 272).

Variable	Categories	N/M	%/IQR
Gender	Male	154	56.6
	Female	118	43.4
Studies Completed	Teacher Training PE	75	27.6
	Physical Activity and Sports Sciences	97	35.7
	Both	89	32.7
Center Environment	Rural	119	43.8
	Urban	153	56.3
Teaching	Primary	111	40.8
	Secondary/High School	161	59.2
Age		44	7
Years of Teaching Experience		18	11

M: Mean; IQR: Interquartile Range.

**Table 2:** Descriptive analysis and differences by sex and center location of the questionnaire items.

Item		Gender			Center Environment		
	Total	Female	Male		Rural	Urban	
	M_e_ (IQR)	M_e_ (IQR)	M_e_ (IQR)	*p*	M_e_ (IQR)	M_e_ (IQR)	*p*
The challenge of being in a regular classroom stimulates the academic development of the student with disabilities.	4 (1)	3.5 (1)	4 (2)	<0.01	4 (1)	4 (2)	<0.01
2. It is easy to maintain order and discipline in an ordinary classroom in which there are disabled students.	3 (3)	3 (2)	3 (3)	0.68	3 (3)	3 (3)	0.94
3. Integration involves group interaction that promotes understanding and acceptance of differences.	5 (1)	5 (0)	5 (1)	<0.01	5 (1)	5 (0.5)	0.11
4. The student with a disability can meets the challenges of the regular classroom on equal terms.	3 (2)	3 (1)	3 (2)	0.40	3 (2)	3 (1)	0.99
5. Integration helps prepare students to live in an integrated society.	5 (0)	5 (1)	5 (0)	<0.01	5 (1)	5 (0)	<0.01
6. I believe that the training of students with disabilities is not the responsibility of the specialists.	4 (2)	4 (2)	4 (2)	0.29	5 (1)	3 (2)	<0.01
7. Those responsible for the development of curricular adaptations should be the educational psychologists.	3 (0)	3 (0.25)	3 (0)	0.04	3 (0)	3 (0)	0.03
8. Those responsible for the implementation and follow-up of the curricular adaptations should be the regular classroom tutors.	4 (2)	4 (2)	4 (2)	0.19	5 (1)	3 (2)	<0.01
9. Specialists and area teachers should assist in the development, implementation and monitoring of curricular adaptations.	4 (2)	5 (2)	4 (2)	0.08	5 (1)	4 (2)	<0.01
10. I believe that the center provides sufficient training for teachers to broaden their knowledge of disability.	3 (1)	3 (1)	4 (1)	<0.01	3 (0)	4 (1)	<0.01
11. The center has sufficient specialized staff to care for students with disabilities.	3 (1)	3 (1)	3 (1)	0.13	3 (1)	3 (1.5)	0.45
12. The organization of the center contemplates the incorporation of supports for the work of the teaching teams.	4 (2)	3 (2)	4 (2)	<0.01	4 (2)	4 (2)	0.01
13. Sufficient space exists to meet the educational needs of students with disabilities.	4 (1)	4 (0)	4 (1)	0.12	4 (0)	4 (1.5)	0.99
14. The center’s infrastructure allows access to and development of the activities of students with disabilities.	4 (1)	4 (1)	4 (1)	0.48	4 (1)	3 (1)	0.02
15. I believe that the use of specialized materials benefits students with disabilities.	5 (1)	5 (0)	5 (1)	0.01	5 (0)	5 (1)	<0.01
16. The extra attention required by students with disabilities shall not be to the detriment of other students.	3 (3)	2 (0)	4 (2)	<0.01	2 (1)	4 (2)	<0.01
17. The integration of students with disabilities shall promote their social independence.	5 (1)	4 (1)	5 (1)	0.01	5 (1)	5 (1)	0.04
18. Integration has a positive effect on the emotional development of the learner.	5 (0)	5 (3)	5 (0)	<0.01	5 (1)	5 (0)	<0.01
19. Students with disabilities behave appropriately in regular classrooms.	4 (2)	4 (1)	4 (2)	<0.01	4 (2)	4 (2)	0.11
20. The social image of students with disabilities as viewed by peers improves through group interaction 2.	5 (1)	5 (0)	5 (1)	<0.01	5 (0)	5 (1)	<0.01
21. In general, integration is a desirable educational practice.	5 (0)	5 (1)	5 (0)	<0.01	5 (0)	5 (0)	0.30
22. It is easy to maintain order and discipline in a regular classroom attended by students with disabilities.	3 (3)	3 (3)	3 (3)	0.51	3 (3)	3 (2)	0.07

Note: M_e_ = median value; IQR = interquartile range. Each score obtained is based on a Likert scale (1–5): 1 is “Strongly disagree” and 5 “Strongly agree”.

**Table 3:** Descriptive analysis of each dimension of the questionnaire.

		Gender			Location of the Center		
Dimensions	Me (IQR)	Female	Male	*p*	Rural	Urban	*p*
Social development	5 (0.4)	4.6 (1.2)	5 (0.4)	<0.01	5 (0.6)	5 (0.4)	<0.01
Classroom climate	3.75 (1.5)	3.5 (1.5)	4 (1.5)	0.51	4.75 (0.5)	3.25 (1.25)	<0.01
Training and resources	3.83 (0.83)	3.5 (0.83)	3.83 (0.5)	0.30	3.83 (0.83)	4 (0.91)	0.23
Performance	3.33 (1)	3 (0.67)	3.66 (1.08)	<0.01	3.33 (0.33)	4 (1.5)	<0.01
Responsibility	3.509 (0.75)	3.25 (0.75)	3.5 (0.25)	0.26	3.50 (0.75)	3.5 (0.62)	0.47

Note: M_e_ = median value; IQR = interquartile range. Each score obtained is based on a Likert scale (1–5): 1 is “Strongly disagree” and 5 “Strongly agree”.

**Table 4:** Correlations between the dimensions and the variable years of experience.

Dimensions	Experience
Social development	0.01
Classroom climate	−0.14 *
Training and resources	−0.02
Performance	0.02
Responsibility	−0.50 **

Note: Correlation is significant at ** *p* < 0.01; * *p* < 0.05. Each score obtained in the dimensions is based on a Likert scale (1–5).

## Data Availability

The datasets used during the current study are available from the corresponding author on reasonable request.
